# Automated Clinical Practice Guideline Recommendations for Hereditary Cancer Risk Using Chatbots and Ontologies: System Description

**DOI:** 10.2196/29289

**Published:** 2022-01-31

**Authors:** Jordon B Ritchie, Lewis J Frey, Jean-Baptiste Lamy, Cecelia Bellcross, Heath Morrison, Joshua D Schiffman, Brandon M Welch

**Affiliations:** 1 Department of Public Health Sciences Medical University of South Carolina Charleston, SC United States; 2 Health Equity and Rural Outreach Innovation Center Ralph H. Johnson Veterans Affairs Health Care System Charleston, SC United States; 3 Université Sorbonne Paris Nord, LIMICS, Sorbonne Université, INSERM, F-93000 Bobigny France; 4 Department of Human Genetics Emory University Atlanta, GA United States; 5 Doxy.me Charleston, SC United States; 6 Division of Pediatric Hematology/Oncology Department of Pediatrics University of Utah Salt Lake City, UT United States; 7 Family Cancer Assessment Clinic, Huntsman Cancer Institute University of Utah Salt Lake City United States, UT United States

**Keywords:** service-oriented architecture, restful API, hereditary cancer, risk assessment, clinical practice guidelines, consumer health informatics

## Abstract

**Background:**

Identifying patients at risk of hereditary cancer based on their family health history is a highly nuanced task. Frequently, patients at risk are not referred for genetic counseling as providers lack the time and training to collect and assess their family health history. Consequently, patients at risk do not receive genetic counseling and testing that they need to determine the preventive steps they should take to mitigate their risk.

**Objective:**

This study aims to automate clinical practice guideline recommendations for hereditary cancer risk based on patient family health history.

**Methods:**

We combined chatbots, web application programming interfaces, clinical practice guidelines, and ontologies into a web service–oriented system that can automate family health history collection and assessment. We used Owlready2 and Protégé to develop a lightweight, patient-centric clinical practice guideline domain ontology using hereditary cancer criteria from the American College of Medical Genetics and Genomics and the National Cancer Comprehensive Network.

**Results:**

The domain ontology has 758 classes, 20 object properties, 23 datatype properties, and 42 individuals and encompasses 44 cancers, 144 genes, and 113 clinical practice guideline criteria. So far, it has been used to assess >5000 family health history cases. We created 192 test cases to ensure concordance with clinical practice guidelines. The average test case completes in 4.5 (SD 1.9) seconds, the longest in 19.6 seconds, and the shortest in 2.9 seconds.

**Conclusions:**

Web service–enabled, chatbot-oriented family health history collection and ontology-driven clinical practice guideline criteria risk assessment is a simple and effective method for automating hereditary cancer risk screening.

## Introduction

### Identifying Patients at Risk of Hereditary Cancer is Challenging

Family health history (FHx) is the most important indicator of the risk of hereditary cancer [1-3]. However, providers have insufficient time to collect and analyze FHx during a patient visit and lack confidence and training in assessing FHx for hereditary cancer risk [4-6]. In addition, the clinical practice guidelines (CPGs) used to assess patient FHx for hereditary cancer risk are numerous and complicated. Many patients with FHx indicative of hereditary cancer risk are unreliably and inaccurately referred for cancer genetic consultation services or are missed altogether [7-9]. Even with accurate FHx collection and assessment, there is a shortage of genetic counselors to meet the needs of cancer genetic consultation services [10,11]. Patients and providers need help in collecting and assessing FHx for hereditary cancer risk to identify patients at risk for earlier counseling and preventive efforts.

### CPGs and FHx Are Important Tools for Identifying Patients at Risk

CPGs contain criteria that amalgamate and organize clinical knowledge relevant to hereditary cancer syndromes. They also define thresholds, curated by experts, based on clinical knowledge for making referral recommendations for cancer genetic counseling and testing [12-15]. There are several organizations that publish these guidelines for various cancer syndromes with varying frequencies, including but not limited to the National Comprehensive Cancer Network (NCCN), the American College of Medical Genetics and Genomics (ACMG), and the US Preventive Services Task Force. Although CPGs are curated by panels of experts and do not necessarily constitute validated tools, they are valuable reference points for considering hereditary cancer risk based on FHx and, if efficiently applied across the patient population, could serve as a valuable indicator of potential risk.

The most useful tool for evaluating whether FHx meets CPG criteria is the family pedigree—a chart with connected squares (males) and circles (females) that depicts family members, their relationships, cancer diagnoses, age, and other relevant information [16]. Risk conveyed to the proband depends on the relationship the proband has with affected and unaffected relatives in their pedigree. The knowledge required to assess CPG criteria can be represented using an ontology. An ontology is a formal, explicit specification of a shared conceptualization [17]. In other words, an ontology is a machine-readable representation of shared knowledge on which thresholds can be evaluated to determine whether FHx meets the criteria for any given patient. Efficiently intersecting family pedigrees and CPG criteria is a necessary step in making timely referral recommendations for cancer genetic consultations.

### Previous Decision Support Tools for Applying CPGs to Patient Data

Over the past several decades, various projects and tools have been built to model and encode CPGs in an effort to increase their value within the clinical workflow. Athena DSS developed at Stanford [18], Asbru developed at Stanford [19], GEM developed at Yale [20,21], GLIF3 [22,23], EON developed at Stanford [24], PROforma developed by John Fox at the Imperial Cancer Research Fund [25], GUIDE [26], Prodigy [27], and, more recently, Sharable Active Guideline Environment (SAGE) [28,29], are all technologies that have been devised to computerize CPGs for hypertension, diabetes, immunization, and others. These tools range widely from XML-based document models to clinical workflow–driven decision support systems designed to formalize CPG knowledge, manage temporal constraints, and integrate with clinical workflows and systems. SAGE, the most recent of these systems, used Protégé [30], an ontology development tool developed at Stanford University, to represent CPG knowledge in ontologies [29].

Ontologies are useful tools for modeling and representing knowledge. More than simple databases, ontologies define concepts and relationships about which inferences can be made beyond logical or statistical measures of the data. Well-known biomedical ontologies that support medical billing, coding, and research include Systematized Nomenclature of Medicine–Clinical Terms [31,32] and the National Cancer Institute Thesaurus [33]. Perhaps the biggest challenge in using ontologies to provide value to clinical care is their size and the processing power required to apply them to patient data. Domain ontologies, as proposed by Musen [34], are designed to overcome this challenge by scoping the ontology to a specific set of concepts and relationships within an application area, such as hereditary cancer. He argues that separating medical knowledge into domain ontologies empowers domain-independent problem solvers, such as software applications in medical informatics, to solve application-level tasks, such as applying CPGs to FHx and recommending genetic counseling.

Unlike the CPG modeling technologies and the ontologies described above, which are designed to be used within clinical workflows and integrate directly with electronic health record (EHR) systems, collecting FHx and assessing hereditary cancer risk are largely agnostic of clinical workflows. Furthermore, FHx in EHR systems is notoriously poor [35,36]. FHx is collectively held by the patient and their family members, which adds to the complication of maintaining accurate FHx within an EHR [37]. Applying CPGs for conditions or domains that depend primarily on data points specific to the patient requires a system that can make the right recommendation at the right time in the clinical workflow based on changing values in a patient’s EHR. However, FHx does not really change a great deal from visit to visit and depends heavily on information the patient may not have during a patient-provider consultation. In addition, as previously pointed out, there is not sufficient time to collect and assess FHx during a patient-provider visit. These challenges necessitate a solution that emphasizes patient involvement and ownership of their family history collection and assessment before visiting their health care provider.

### Web Application Programming Interfaces and Chatbots Increase Patients’ Access to CPG Recommendations

Chatbot-oriented FHx collection and ontology-driven CPG risk assessment implemented in a web service architecture have the potential to empower patients through a simple and effective mechanism for automating initial FHx collection and risk assessment. In previous studies, we demonstrated the utility of collecting FHx using chatbots and web services, the most recent of which engaged >10,000 individuals in collecting and assessing their FHx [38,39]. Research has shown that although conversational chatbot agents can take a little longer to interact with, users reported higher overall satisfaction, perceived usefulness of the system, perceived quality of information collected, and significantly better interface quality, with 3 out of 4 users preferring chatbots to traditional data collection methods [40,41]. The chatbot we built for the system is a workflow-driven chatbot that follows a branching logic strategy for optimal user experience [39,42]. The observed participation in collecting FHx using chatbots is evidence that patients with cancer in their families are motivated to learn about their risk. Once patients collect their FHx using the chatbot, they only need access to CPG criteria for initial risk assessment that does not require the time and attention of a trained professional.

Access is best provided to formalize CPG knowledge for risk assessment using web application programming interfaces (APIs) that can receive electronic FHx data and return CPG recommendations. Web APIs, or representational state transfer APIs, form the underpinnings of modern web development by providing access to data, processes, and information on the web in a general, scalable, and secure manner through the browser [43]. By combining chatbots and ontological representations of CPGs for FHx with web APIs, patients can collect their FHx and receive CPG recommendations from the comfort of their own home with their family members and share their results with their provider at a future provider consultation.

The objectives of our study are to collect and store FHx in an electronic format, organize CPGs into a knowledge representation that can be applied to the FHx, and design a system that can assess FHx using CPG criteria and return the relevant recommendations using web APIs. This paper describes the ontological representation of hereditary cancer CPGs from NCCN and ACMG and the system that applies the CPG ontology to patient FHx to determine whether cancer genetic consultation should be considered. This paper will help biomedical informaticists and web application developers understand how to automate the application of domain ontologies to patient data using ontology programming interfaces (OPIs) and web APIs.

## Methods

### Hereditary Cancer CPG Ontology

#### Overview

The hereditary cancer CPG ontology was developed by JBR and reviewed by LF. We selected criteria from the ACMG and NCCN hereditary cancer CPGs for the most prevalent cancer syndromes. These criteria outlined the domain knowledge necessary to create an ontological representation of CPGs and write rules in the ontology that represent the CPG criteria. Ontologies were developed using Python (version 3.7), the Protégé ontology editor (version 5.2.0), and the Owlready2 OPI (version 2.21), which includes a modified version of the HermiT Reasoner developed by the Department of Computer Science at the University of Oxford [44]. The ontology was designed to represent all possible states for patient FHx according to the CPGs as efficiently as possible. We used Owlready2 to dynamically generate and modify the ontology in Python using JSON data structures. The system is open source and available on Bitbucket [45].

#### Representing CPGs Using Ontologies

**Figure 1:**
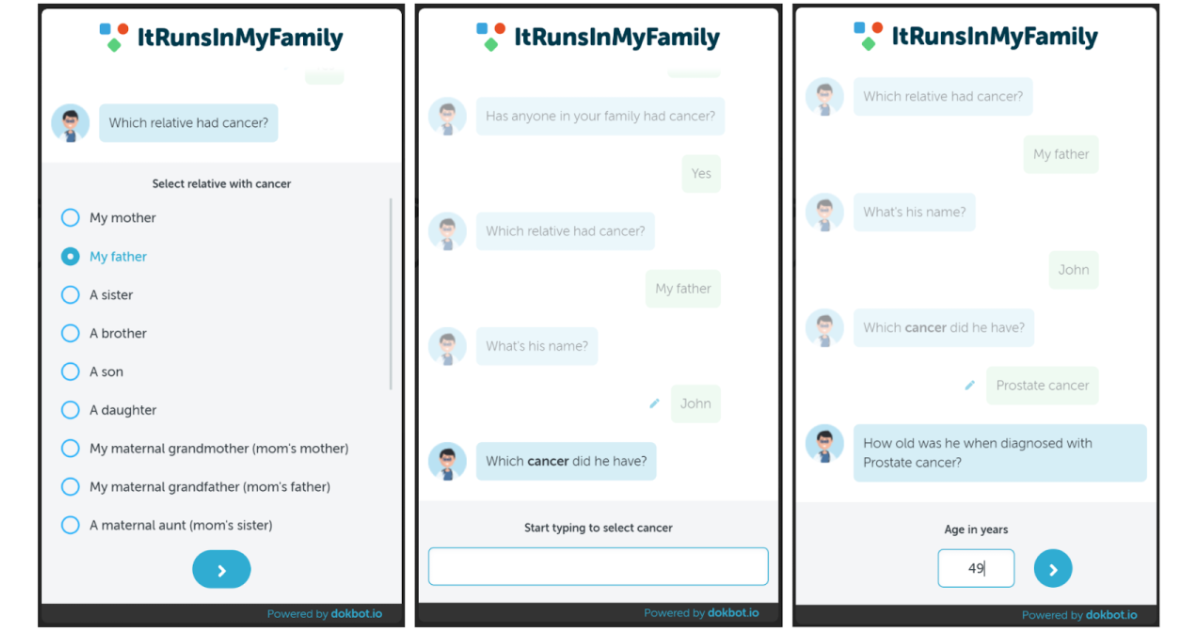
The chatbot collects family health history (FHx) relevant to CPG ontology logic. In this example, the patient enters values for an FHx where the father has prostate cancer before the age of 55 years. The workflow will also collect the same data for all other family members. Per the example in the text, if both father and a brother of the proband have prostate cancer before the age of 55 years, they would meet the ACMG criterion ≥2 cases of prostate cancer diagnosed at age ≤55 years in close relatives. ACMG: American College of Medical Genetics and Genomics; CPG: clinical practice guidelines.

Hereditary cancer domain knowledge from the CPGs is defined in the ontology using concepts, properties, and individuals. Relationships between concepts are defined in the ontology by using Resource Description Framework triples and equivalency classes to represent CPG criteria that can be applied to FHx instantiated as individuals in the ontology. For example, consider a family history where the proband has a father and a brother both diagnosed with prostate cancer before the age of 55 years. When the patient engages the chatbot, the family member workflow will ask which family members had cancer and what age they were diagnosed (Figure 1). On the basis of the answers to these questions, which directly tie to the ontology logic described below, this proband is considered at risk and should consider a cancer genetic consultation based on the following criterion in ACMG: ≥2 cases of prostate cancer diagnosed at age ≤55 years in close relatives. This criterion is modeled in the ontology in two separate subclasses of ACMGProstatePatient:



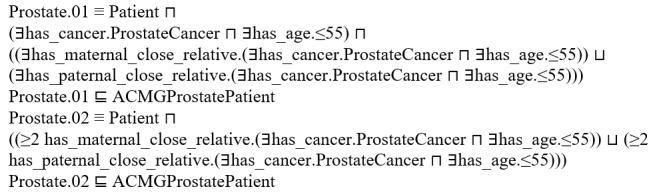



Prostate.01 accounts for the cases where the proband and a close family member have prostate cancer diagnosed before the age of 55 years. Prostate.02 accounts for cases where the proband does not have cancer but 2 close family members have prostate cancer diagnosed before the age of 55 years. An important rule that applies to all criteria is that rules involving ≥1 family member must be on the same side of the family to truly evaluate hereditary patterns. The aforementioned example rules account for this by considering that the relationships *has_maternal_close_relative* and *has_paternal_close_relative.* Together, the aforementioned rules represent the prostate criterion that our example proband meets. When the reasoner classifies the proband as a subclass of the ACMGProstatePatient, the system knows to recommend a genetic cancer consultation for the proband. Equivalencies such as Prostate.01-02 capture CPG criteria and are the crux of automated identification of patients at risk of hereditary cancer. A list of all criteria implemented from ACMG and NCCN can be found in Tables S1 and S2 in the Multimedia Appendix 1.

#### Ontology Construction

To successfully apply the CPG ontology to patient data, we recognized that certain design patterns were necessary to ensure reasonable processing time and out-of-the-box application of the HermiT Reasoner. First, only CPG knowledge concepts necessary for applying the CPG criteria should be included in the CPG domain ontology to prevent bloat and ensure acceptable reasoning times with the HermiT Reasoner. Therefore, concepts related to treatment, for example, are not included. Second, the CPG criteria rules should be contained as subclasses of the *Patient* class, thereby ensuring that after a patient’s FHx is instantiated in the ontology and the reasoner has completed reasoning, the patient has been reclassified within the ontology under *Patient* subclasses that correspond to the CPG criteria met by their FHx, for example, ACMGProstatePatient. The result is a lightweight, patient-centric domain ontology that is readily adapted to run inside a web API.

Ontology construction is an iterative process that relies heavily on the CPGs to determine concepts, relationships, and equivalencies to be defined in the ontology. Throughout the development process, the criteria interpretations in the ontology equivalency classes were reviewed by a genetic counselor (CB) and an oncologist (JDS). As new classes were added to the ontology, test cases were created to ensure that the equivalency classes worked as expected.

FHx assessment depends on how many family members on one side of the family are diagnosed with certain combinations of cancers at or before specific ages in the presence of specific disease factors. The thresholds defined by the CPGs are minimum thresholds that require frequent use of the cardinality restriction *MIN*. Ontological reasoning with cardinality restrictions is complex and time consuming. Other methods, such as SPARQL Protocol and RDF Query Language (SPARQL) queries, do not handle cardinality restrictions easily. Handling cardinality restrictions with SPARQL is difficult as if one considers a cardinality restriction with a cardinality of n, one needs to (1) search the n relations, (2) verify that they are all distinct, and (3) remove duplicates (eg, if n=2 and one finds the a,b relation, then b,a should not be considered as a distinct result). This typically requires many triples in SPARQL, especially if the value of the cardinality restriction is complex (for example, another restriction), as in that case, it must be copied n times in the SPARQL query. Another option is to use a *GROUP BY* statement in SPARQL. However, this allows only a single cardinality restriction. If there is ≥1 such restriction, it would require us to run multiple separate queries and then take the union of their results [46]. Thus, we used an ontological definition as we had to rely heavily on the MIN cardinality restriction to implement the criteria. To keep reasoning times down, we frequently used ≥1 equivalency class to represent a single CPG criterion.

#### Owlready2 and Ontology-Oriented Programming

Owlready2 is a lightweight Python library designed to programmatically create and edit ontologies [47]. By using Python’s inherent hierarchical class structure, Owlready2 provides an intuitive OPI for ontology-oriented programming. In addition, Owlready2 is able to bind specific programmatic functionality to ontology concepts by declaring Python functions directly within ontology classes. This ability makes it possible to treat ontology classes such as objects in object-oriented programs. Web APIs are generally built using object-oriented programming development patterns and often control interactions with relational databases through an object relational mapper (ORM) [48,49]. ORMs are the link between web APIs and relational databases that provide access and management of data in the database via the web API. We used Owlready2 as a key resource in developing the CPG ontology but, more importantly, as a kind of ORM for interfacing with the ontology to provide our web API access to the knowledge in the ontology for FHx cases.

#### System Evaluation and Testing

**Figure 2:**
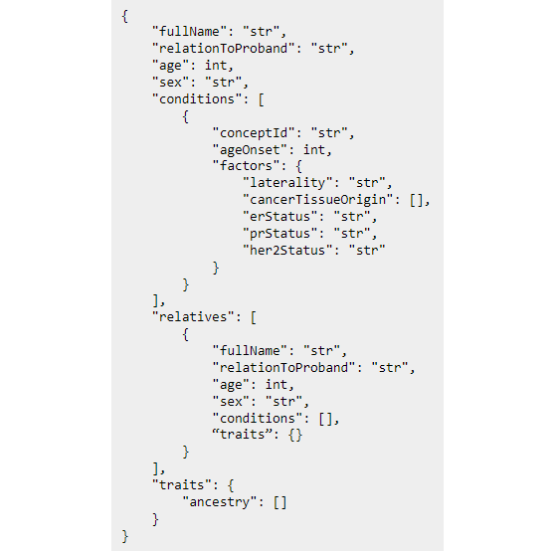
Example JSON family health history (FHx) format, JSON is a ubiquitous data structure for web development based on key value pairs. Each object in the relatives list represents a family member with the exact same FHx format as the proband. This example only shows factors for breast cancer.

We created 192 test cases—at least one test per CPG criterion—to evaluate the system and ensure equivalency classes performed as expected. Each test case had a target and a payload. The target is the correct recommendation in the CPG ontology, and the payload is the test case FHx. The FHx in the payload adheres to the same JSON schema as the FHx received by the chatbot (Figure 2). The creation of test cases was heavily driven by the criteria for which the test was written. Each test case was built to reflect combinations of family cancer diagnoses, disease factors, and ages of onset to trigger the target CPG criterion.

### System Architecture

#### Overview

Patient FHx collected by the chatbot [39] is sent to a web API (FHx API) that manages access to the CPG ontology. The FHx API is responsible for instantiating the FHx using the CPG ontology, initiating the HermiT Reasoner to apply CPG criteria, and retrieving final recommendations. The HermiT Reasoner is a state-of-the-art ontology reasoner that is packaged with most ontology development resources such as Protégé and Owlready2 [50]. The FHx API then sends the results to another web API (report API) that packages the information into a PDF report. The report API is capable of sending the report to the patient or to the patient’s provider.

#### FHx API Components

**Figure 3:**
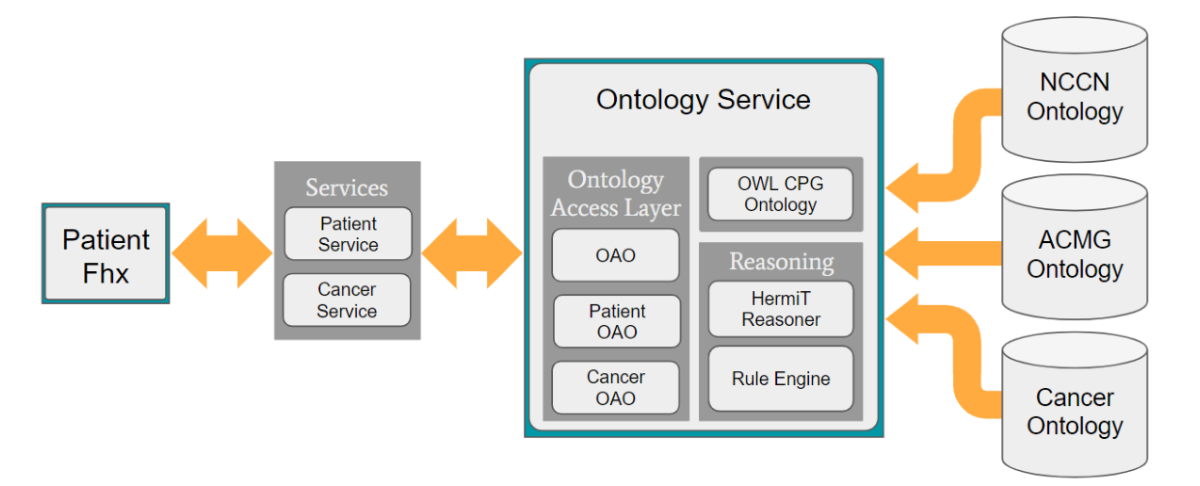
System architecture. Patient family health history (FHx) is received by the system; services use the ontology service to model patient data using the CPG ontology, perform reasoning, and make recommendations. ACMG: American College of Medical Genetics and Genomics; CPG: Clinical practice guidelines; NCCN: National Comprehensive Cancer Network; OAO: ontology access object.

The FHx API has an ontology access object (OAO) layer, a service layer, and a reasoning layer (Figure 3). The OAOs coordinate access to the ontology for all other API components. The ontology service is the most important part of the FHx API and is responsible for providing access to the CPG ontology, all OAOs, and the HermiT Reasoner. There are two other main services: Patient service and Cancer service. These 2 services are specifically named after the Patient and Cancer classes in the CPG ontology and have corresponding OAOs. Importantly, they each have access to the ontology service to coordinate with their respective OAOs to instantiate FHx using the ontology and initiate reasoning. In addition, they use their OAOs to retrieve recommendation results after the HermiT Reasoner completes.

#### Instantiating Patient Data With Services and OAOs

Owlready2 allows Python functions to be declared within ontology classes, enabling object-oriented programming methods to be used to instantiate FHx using the CPG ontology. The OAO layer makes the most use of this by defining the methods for setting relationships and other important properties necessary for instantiating the FHx in the ontology. Each service in the service layer has an associated OAO wherein all Python functions that immediately access the ontology reside. For example, the Patient service receives patient FHx in JSON format and relies on the Patient OAO to add family members to the ontology as individuals, set family relations, and retrieve recommendations. An example patient FHx JSON format can be viewed in Figure 2. Separating service logic from OAOs isolates interactions with the ontology and emulates a well-established pattern of developing traditional web APIs where access to relational databases is encapsulated within database access objects. Once the patient FHx is instantiated using the CPG ontology, it is ready for the HermiT Reasoner to apply CPG criteria.

#### Reasoning and Retrieving Results With OAOs

After the patient’s FHx has been instantiated by creating individuals in the ontology to represent the family members and their respective conditions, the ontology service calls the reasoning layer. Owlready2 uses the HermiT Reasoner to execute previously defined equivalency classes within the ontology that contains CPG criteria. Patient FHx instantiated within the ontology is reclassified accordingly to indicate which CPG criteria they meet, if any. Once the reasoning is completed, the service layer accesses the reclassified FHx from the ontology using OAOs and returns CPG-based recommendations (Figure 3). Importantly, the CPG ontology is reloaded for each FHx it evaluates to ensure that FHx from previous probands has been removed.

## Results

### Hereditary Cancer CPG Ontology

#### Overview

**Figure 4:**
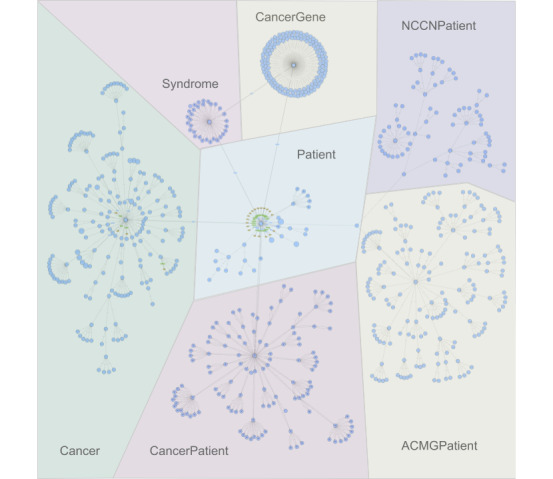
Ontology graph produced using WebVOWL. The Patient class is the central feature of the ontology and is linked to the *Cancer, Syndrome,* and *CancerGene* classes by the properties *has_cancer, has_syndrome,* and *has_mutation_in,* respectively. *Ancestry, CancerTissueOrigin, DiseaseFactor, Histology, HormoneStatus, Laterality, Polyp, Sex,* and *Trait* are concentrated around the Patient class. The right side of the graph represents the NCCNPatient class and the ACMGPatient class and their respective subclasses. ACMG: American College of Medical Genetics and Genomics; NCCN: National Comprehensive Cancer Network.

Using Python, Owlready2, and Protégé, we generated a hereditary cancer ontology with 758 classes, 20 object properties, 23 datatype properties, and 42 individuals and visualized it using WebVOWL [51] to produce a graph with 781 nodes and 1015 edges (Figure 4). The blue circles represent classes in the ontology class hierarchy, the blue boxes on lines between concepts represent object properties, and the green boxes on lines between the yellow boxes represent data properties and data types, respectively. The parent classes in the ontology class hierarchy are *Ancestry*, *Cancer*, *CancerGene*, *CancerTissueOrigin*, *DiseaseFactor*, *Histology*, *HormoneStatus*, *Laterality*, *Patient*, *Polyp*, *Sex*, *Syndrome*, and *Trait* and include 44 cancers, not including subtypes, 144 genes, 73 criteria from ACMG, and 40 criteria from NCCN. Static individuals in the ontology are represented by increasing the area of the concept they belong to in the graph. *Ancestry* (10 static individuals) is the largest, followed by *CancerTissueOrigin* (8 static individuals)*, Histology* (6 static individuals)*, HormoneStatus* (6 static individuals)*, Trait* (4 static individuals)*, Sex* (3 static individuals)*, Laterality* (3 static individuals)*,* and *DiseaseFactor* (2 static individuals).

#### Patient Class

The most important class in the ontology is that of the *Patient* and every family member, including the proband, and is instantiated as an individual of the Patient class when a patient’s FHx is processed. The most important subclasses of the Patient class are *CancerPatient* and *PatientWithRecommendations*. *CancerPatient* is used to define the proband and their family members in terms of the cancers they have, and *PatientWithRecommendations* is the parent class of *ACMGPatient* and *NCCNPatient classes*. All CPG criteria are housed in the equivalency subclasses of *ACMGPatient* and *NCCNPatient* and rely on the equivalency classes in *CancerPatient* and *Cancer* to evaluate the CPG criteria. The CPG criteria implemented from ACMG and NCCN can be found in Tables S1 and S2 in the Multimedia Appendix 1. In Figure 4, *Ancestry*, *Polyp, Sex,* and *Trait* are also inside the *Patient* block. Although they are not strictly subclasses of the *Patient* class, they represent small clusters of the ontology that are directly related to the *Patient* class*.*

The *PatientWithRecommendations* class is the parent class of all the guidelines implemented by the ontology. *ACMGPatient* and *NCCNPatient* represent 2 isolated clusters in the ontology that encapsulate separate but very similar hereditary cancer guidelines. Each leaf node in *ACMGPatient* and *NCCNPatient* represents a CPG criterion used to evaluate patient FHx. Breast, ovarian, pancreatic, colorectal, and endometrial cancer guidelines are implemented for both ACMG and NCCN, along with guidelines specific to Li–Fraumeni syndrome (LFS) and Lynch syndrome (LS). In addition, brain, gastric, melanoma, prostate, renal, and thyroid cancer guidelines are implemented for ACMG. This accounts for the relative size difference between the *ACMGPatient* and *NCCNPatient clusters.*

#### Cancer

*Cancer* is the next most important class in addition to the *Patient* class in the ontology class hierarchy for evaluating patient FHx. A total of 44 cancers (83 including subtypes) are represented in the *cancer* block in Figure 4. Although not all of them are immediately pertinent to hereditary cancer CPGs, it is important to include them in an accurate patient FHx. The most frequently used cancers by the CPGs are breast, ovarian, colorectal, endometrial, and those related to LFS and LS. In Figure 4, *CancerTissueOrigin, DiseaseFactor, Histology, HormoneStatus,* and *Laterality* are also inside the *cancer* block and are represented by static individuals. *CancerTissueOrigin* is specific to where the cancer originated in a patient, for example, *ductal* and *lobular* for breast cancer; *DiseaseFactor* includes factors for different cancers, for example, *mmr_stable; Histology* represents different histologies, most notably for kidney cancer, for example, *clear_cell* or *collecting_duct; HormoneStatus* represents positive or negative estrogen, progesterone, or human epidermal growth factor 2 for breast cancer, for example, *er_positive;* and *Laterality* indicates one or both sides of the body, most notably with regard to breast cancer, for example, *bilateral*.

#### Syndrome and CancerGene

The *Syndrome* and *CancerGene* clusters contain 32 and 141 concepts, respectively. The syndromes included in the ontology are curated directly from the list of syndromes in the ACMG CPG [14], and the cancer genes come from reviewing ACMG and NCCN CPGs as well as genetic tests from well-known cancer testing companies such as Myriad Genetics, ARUP Laboratories, GeneDx, and others. The most important syndromes for evaluating the CPG criteria are LFS and LS along with their associated gene mutations: TP53 for LFS and EPCAM, MLH1, MSH2, MSH6, and PMS2 for LS.

### System Architecture

All 192 test cases were built to ensure the accuracy of the implemented criteria completed in 14 minutes and 47 seconds. The longest time a test took to complete was 19.6 seconds, the shortest was 2.9 seconds, and the average was 4.5 (SD 1.9) seconds. The reasoning time varied with the number of family members and the combination of cancers and disease factors present in the FHx. The response time of the entire system (chatbot–report) depends on the number of family members and the total number of cancer diagnoses in the FHx. A typical FHx case with 3 to 5 cancers and 20 to 25 family members takes approximately 20 to 40 seconds. However, various combinations of cancers and family members can take as little as 8 seconds or as long as 5 minutes. The system is asynchronous; it can process FHx for multiple probands at a time (2×CPU count+1 threads) and sends PDF reports to the proband once the reasoner completes and the PDF is rendered. At the end of the assessment, the chatbot notifies the proband that as soon as their FHx is finished processing, the PDF report will be sent to the email address they provided.

In separate studies, we report on proband recruitment and FHx collection [39] and compare the results of ACMG and NCCN criteria applied to the FHx by the system for 4915 probands who have collected their FHx using the system and received a report [52]. Of those, 2221 probands met the criteria, and 2694 did not meet the criteria. Breast and ovarian cancer guidelines were the most consistent, and colorectal and endometrial guidelines were the most disparate across the ACMG and NCCN. Of all probands who did not meet the criteria, 90.6% had cancer in their FHx. In an additional study, we compared the referral patterns for genetic counselors, oncologists, and primary care providers to determine the level of concordance with the CPG criteria implemented by ItRuns [53]. Oncologists and primary care providers had consistently lower rates of concordance with CPG criteria, especially for probands whose FHx triggered the CPG criteria, indicating an immediate opportunity for the system to help frontline care providers identify patients at risk if the system were implemented across the primary care population. Genetic counselors had very high concordance with CPG criteria, especially for probands who met the criteria, and the ontology classification of the system had high concordance with genetic counselors, indicating tight coupling between the CPG recommendations and genetic counselors’ professional assessments. The system has strict adherence to CPG criteria and has the potential to reduce human error in FHx collection and risk assessment.

## Discussion

### Principal Findings

We curated an ontology using Owlready2 and Protégé and developed a web system to apply CPG criteria to patient FHx and identify probands who should consider a cancer genetic consultation. Intersecting the CPG ontology with patient FHx using traditional web development strategies provides patients with access to evidence-based recommendations without requiring the initial time and effort of trained professionals.

#### Hereditary Cancer CPG Ontology

##### Potential Impact for Identifying Patients at Risk

Identifying patients at risk of hereditary cancer is a multilayered and highly nuanced challenge. Providers lack time and training for collecting and assessing hereditary cancer risk during patient visits; genetic counselors trained in FHx collection and assessment are in short supply; and patients lack the expertise to interpret CPG criteria for themselves. The end goal is to get patients whose FHx meets the CPG criteria in front of genetic counselors as soon as possible for preventive actions to have the maximum impact on patient outcomes. Chatbots simplify the process of collecting FHx, do not require trained professionals, and are designed for a positive user experience. FHx collected by chatbots is by default in electronic format and ready for analysis. Ontologies are validated tools for modeling CPGs and, with the help of Owlready2, can be accessed using web APIs to assess FHx for hereditary cancer risk. The results can be shared with the patient and the provider before a consultation, effectively removing barriers to referring patients whose FHx indicates hereditary cancer risk to meet with genetic counselors for a cancer genetic consultation.

##### Owlready2 and Ontology Development

Not all ontologies are naturally adaptable to applying CPG criteria to patient data, and there is certainly ≥1 ontology formalism that would satisfy the needs of the hereditary cancer CPG ontology. Ontologies, especially biomedical ontologies, are generally organized and optimized for dictionary-like functions such as looking up information and modeling relationships as closely as possible to the real world. Understandably, this ontology development objective sometimes leads to very large biomedical ontologies, such as Systematized Nomenclature of Medicine–Clinical Terms and the National Cancer Institute Thesaurus, which require impractical processing power for the HermiT Reasoner to reason with. However, more importantly, as these ontologies are not modeled with the patient as a central concept, the HermiT Reasoner is not sufficient to apply CPG criteria out of the box without additional work performed by supporting functions in the FHx API. A lightweight, patient-centric, domain-specific ontology that is small enough to run inside a web API is crucial to quickly apply CPG criteria to FHx. Importantly, a proband’s risk depends on whether first-degree relatives meet the CPG criteria. Therefore, each family member in the ontology can be instantiated as an individual of the *Patient* class and be classified according to the CPG equivalency classes.

#### System Architecture

##### Domain Ontologies and Service-Oriented Architecture

Lightweight, patient-centric domain ontologies align with a modular service-oriented architecture (SOA) approach to applying CPGs to patient FHx. SOA is a web development architectural pattern that allows small applications to work together over a network to achieve an overall end goal. For example, the chatbot is one such service, and once a patient’s FHx is collected, it sends the FHx to the FHx API, which is another service. Once the FHx API has applied CPGs to the patient FHx, it sends the results to another service to create and send the PDF report. The CPG ontology is a component accessed by the FHx API service. As it is lightweight and patient-centric, it can be applied in a modular fashion. For example, the isolation of *ACMGPatient* and *NCCNPatient* in the hierarchy in the ontology (Figure 4) enables a plug-and-play style of applying CPGs to patient FHx. All other classes in addition to *ACMGPatient* and *NCCNPatient* in the hereditary cancer CPG ontology, along with properties and individuals in Figure 4, represent a common set of knowledge needed for evaluating both the ACMG and NCCN criteria. This common set of knowledge between ACMG and NCCN allows *ACMGPatient* and *NCCNPatient* to be executed independently of each other by loading 2 separate, smaller CPG ontologies—an ACMG CPG ontology and an NCCN CPG ontology—that depend on the same domain knowledge. SOA applies these 2 smaller ontologies to patient FHx in parallel and synthesizes their results together at the end, thereby decreasing the time to complete the reasoning with the HermiT Reasoner and sending results to the report service. The system works asynchronously, and the chatbot informs patients that when reasoning is complete, they will be emailed a PDF report with their results. In theory, this approach could be applied to any combination of ontologies for ≥1 domain, as long as the ontologies are sufficiently small and patient-centric.

##### System Results Compared With Professional Assessments

Automating FHx and hereditary cancer risk assessment reduces irregularities in data collection and the application of CPG criteria. Standardizing FHx collection and the application of CPG criteria is an important step in helping to consistently identify patients at risk of hereditary cancer. It is true that the system is rigidly tied to the CPG criteria, and CPGs are not validated resources. However, CPGs are very valuable, empirically derived benchmarks curated by experts, which could provide an initial screen that is largely currently missing on the frontlines of care. The system is not intended to replace professional assessments but rather complement them. Indeed, we found in our comparison of genetics and non–genetics providers’ professional assessment cases that the CPGs applied by the system were discordant with provider recommendations. These cases were often cases where FHx fell just short of meeting a criterion’s threshold for cancer cases in the family or age of diagnosis. The initial assessment performed by the system is designed to alert patients and physicians when a professional assessment is warranted strictly according to the CPG criteria. Our preliminary data comparing genetics and non–genetics professionals’ concordance with CPGs indicates that non–genetics professionals (primary care physicians and oncologists) unsurprisingly have low concordance, and genetics professionals have high concordance with CPG criteria [53]. The observed high concordance with CPG criteria for genetics professionals and the system is evidence that primary care population-wide application of the system could reduce human error in CPG criteria application to FHx and support primary caregivers in identifying patients at risk of hereditary cancer. In addition, by having formalized actionable rules, cases where providers are discordant with CPGs can be identified as places where the guidelines can potentially be improved. Such improvements could result from human judgment and intuition, which interact with formalized logic encoded within the system.

Importantly, the system is intended to be an initial screen and is not intended to replace professional assessments. In fact, the system is designed to augment the genetics and non–genetics professionals’ capacity to collect and assess FHx risk for hereditary cancer by recommending patients whose FHx meets CPG criteria to seek a cancer genetic consultation. Although the system rigidly adheres to nonvalidated CPG guidelines and might at times be discordant with health care providers’ professional assessments, the broad application of the system to the primary care population would increase the identification of patients who do meet criteria dramatically from the current state of hereditary cancer risk assessment. Our preliminary data show that genetic counselors have very high concordance with CPG criteria for FHx that does, in fact, meet CPG criteria and non–genetics professionals do not. The notion of false positives and false negatives in this context is nuanced. As the CPGs are not validated tools but are curated by panels of experts, in the case that a health professional is discordant with the CPG, it is difficult to determine who is correct. In the event the system provides evidence-based recommendations for a cancer genetics consultation, and the health care professional disagrees, at least the patient and provider have increased awareness of the patient’s FHx risk status for hereditary cancer. In the event the system does not provide an evidence-based recommendation for a cancer genetic consultation, but the provider would recommend counseling, the patient still collects FHx and can show it to their provider in future appointments. In either case, the purpose of the system is fulfilled by collecting FHx and applying CPG criteria to assess risk and raise awareness. More work needs to be conducted to implement the system in a broader clinical context; however, strict adherence to CPG criteria would definitely be a step forward from the current lack of application of any FHx risk assessment at the primary care level [52-54].

### Limitations

The HermiT Reasoner is a very efficient ontology reasoner and is capable of reasoning over large ontologies. However, even small ontologies take several seconds to reason with, and most web applications typically adhere to subsecond response times for optimal user experience. The computation time required to apply the HermiT Reasoner to patient FHx necessitates an asynchronous experience where patients receive an email with their PDF report 30 seconds to a minute after completing the chatbot questions. Although this is workable, ideally, patients would receive immediate feedback upon completing their FHx collection. We developed a custom rule engine for applying CPG criteria without using the HermiT Reasoner, which increased the processing time substantially. However, more work is required to make it generally applicable across all domains.

### Comparison With Prior Work

The prior systems we chose for comparison in this study were systems designed specifically to computerize a wide range of CPGs for broad application and use. The system described in this paper similarly outlines an approach for automating the application of rule-based CPGs. Hereditary cancer was selected to demonstrate the effectiveness of the approach but is intended to be applied to other use cases. There are a number of other technologies designed to encode CPGs for various clinical decision support purposes such as Athena DSS [18], Asbru [19], GEM [20,21], GLIF3 [22,23], EON [24], PROforma [25], GUIDE [26], Prodigy [27], and SAGE [28,29]. However, these systems are older, difficult to access, and more oriented toward integrating with clinical workflows. Clinical workflow integration primarily supports clinicians and is generally less accessible to patients. DESIREE (Decision Support and Information Management System for Breast Cancer) is a more recent example of such a solution that uses ontological reasoning to support CPG application to patients with breast cancer in clinical settings to help tumor boards develop care plans [55-57]. Similar to our system, DESIREE and SAGE used Protégé to develop domain ontologies to computerize CPGs.

A system developed by Abidi [58] has some parallels with our approach. They built a system for a breast cancer follow-up CPG that used GEM to computerize the CPG criteria. Their approach is similar in the use of ontologies to computerize CPGs and execute reasoning to obtain recommendations; however, the methods and applications are quite different. Their system was designed for clinicians to author rules based on the CPGs using GEM, whereas we built a system that outlines a replicable development pattern designed for application in a modern web development environment.

The solution we built is distinctly different in that it applies widely accepted web development best practices to ontology curation and application, focuses on empowering patients with CPG-driven recommendations instead of integrating with provider workflows, and uses a chatbot optimized for mobile devices that simplifies FHx collection and seamlessly interoperates with ontology-driven risk assessment. In addition, our codebase is open source and available on Bitbucket [45].

### Conclusions

Combining web APIs, chatbots, ontologies, and hereditary cancer CPGs has the potential to identify patients at risk of hereditary cancer based on patient FHx more efficiently. Patients can collect and receive CPG-driven insights about their FHx before seeing their health care provider, thereby removing the burden of initially collecting and assessing FHx with trained professionals. Ontology-assisted CPG-driven recommendations serve as a temperature check, offering an initial indication of whether patients and providers should consider a cancer genetic consultation based on FHx. Earlier identification of patients at risk for hereditary cancer based on their FHx will result in earlier preventive actions for better outcomes.

## Appendix

Multimedia Appendix 1 Clinical practice guidelines and ontology concepts.

## Abbreviations

ACMG: American College of Medical Genetics and Genomics
API: application programming interface
CPG: clinical practice guideline
DESIREE: Decision Support and Information Management System for Breast Cancer
EHR: electronic health record
FHx: family health history
LFS: Li–Fraumeni syndrome
LS: Lynch syndrome
NCCN: National Comprehensive Cancer Network
OAO: ontology access object
OPI: ontology programming interface
ORM: object relational mapper
SAGE: Sharable Active Guideline Environment
SOA: service-oriented architecture
SPARQL: SPARQL Protocol and RDF Query Language
