# Supplementary material for: Automated Clinical Practice Guideline Recommendations for Hereditary Cancer Risk Using Chatbots and Ontologies: System Description
Source: JMIR Cancer. 2022 Jan 31;8(1):e29289. doi: 10.2196/29289 (PMC8845001; doi:10.2196/29289)
Supplement: Multimedia Appendix 1 [file cancer_v8i1e29289_app1.docx]

## Multimedia Appendix 1: Clinical Practice Guidelines and Ontology Concepts

Table S1. ACMG guidelines and criteria implemented: Criteria from brain, breast, colorectal, endometrial, gastric, leukemia, melanoma, ovarian, pancreatic, prostate, renal, and thyroid cancer CPGs from ACMG were represented with equivalency classes in the ontology.

| ACMG Hereditary Cancer CPGs implemented in CPG Ontology | |
| --- | --- |
| Brain |  |
|  | Brain tumor dx at age <18 if any of the following criteria are met:  –Family history of LS-associated cancer  –Second primary cancer  –Sibling with a childhood cancer |
|  | Brain tumor and two additional cases of any LS-associated cancer (Table 6) in the same person or in relatives |
|  | Brain tumor and one additional LFS tumor (Table 5) in the same person or in two relatives, one dx at age ≤45 |
|  | Astrocytoma and melanoma in the same person or in two FDRs |
|  | Medulloblastoma and ≥10 cumulative adenomatous colon polyps in the same person |
| Breast cancer, female |  |
|  | Breast cancer dx at age ≤50 |
|  | Triple-negative breast cancer dx at age ≤60 |
|  | ≥2 primary breast cancers in the same person |
|  | Ashkenazi Jewish ancestry and breast cancer at any age |
|  | ≥3 cases of breast, ovarian, pancreatic, and/or aggressive prostate cancer in close relatives, including the patient |
|  | Breast cancer and one additional LFS tumor (Table 5) in the same person or in two relatives, one dx at age ≤45 |
|  | Breast cancer and ≥1 PJ polyp in the same person |
|  | Lobular breast cancer and diffuse gastric cancer in the same person |
|  | Lobular breast cancer in one relative and diffuse gastric cancer in another, one dx at age <50 |
| Breast cancer, male |  |
|  | Single case present |
| Colorectal cancer |  |
|  | Colorectal cancer dx at age <50 |
|  | Colorectal cancer dx at age ≥50 if there is a FDR with colorectal or endometrial cancer at any age |
|  | Synchronous or metachronous colorectal or endometrial cancers in the same person |
|  | Colorectal cancer showing mismatch repair deficiency on tumor screening |
|  | Colorectal cancer and two additional cases of any LS-associated cancer (Table 6) in the same person or in close relatives |
|  | Colorectal cancer and one additional LFS tumor (Table 5) in the same person or in two relatives, one dx at age ≤45 |
|  | Colorectal cancer with ≥10 cumulative adenomatous colon polyps in the same person |
| Endometrial cancer |  |
|  | Endometrial cancer dx at age <50 |
|  | Endometrial cancer dx at age ≥50 if there is a FDR with colorectal or endometrial cancer at any age |
|  | Synchronous or metachronous colorectal or endometrial cancer in the same person |
|  | Endometrial cancer showing mismatch repair deficiency on tumor screening |
|  | Endometrial cancer and 2 additional cases of any LS-associated cancer (Table 6) in the same person or in close relatives |
| Gastric cancer |  |
|  | ≥2 cases of gastric cancer, one dx at age <50 in close relatives |
|  | ≥3 cases of gastric cancer in close relatives |
|  | Diffuse gastric cancer dx at age <40 |
|  | Diffuse gastric cancer and lobular breast cancer in the same person |
|  | Diffuse gastric cancer in one relative and lobular breast cancer in another, one dx at age <50 |
|  | Gastric cancer and 2 additional cases of any LS-associated cancer (Table 6) in the same person or in close relatives |
| Leukemia |  |
|  | Leukemia dx at age <18, if any of the following criteria are met:  –Family history of LS-associated cancers  –Second primary cancer  –Sibling with a childhood cancer |
|  | Leukemia and one additional LFS tumor (Table 5) in the same person or in 2 close relatives, one dx at age ≤45 |
| Melanoma |  |
|  | ≥3 cases of melanoma and/or pancreatic cancer in close relatives |
|  | ≥3 primary melanomas in the same person |
|  | Melanoma and pancreatic cancer in the same person |
|  | Melanoma and astrocytoma in the same person or in 2 FDRs |
| Ovarian/Fallopian tube/  primary peritoneal cancer |  |
|  | Single case present in the patient or a FDR |
| Pancreatic cancer |  |
|  | Pancreatic cancer dx at any age, if any of the following criteria are met:  –≥2 cases of pancreatic cancer in close relatives  –≥2 cases of breast, ovarian, and/or aggressive prostate cancer in close relatives  –Ashkenazi Jewish ancestry |
|  | Pancreatic cancer and ≥1 PJ polyp in the same person |
|  | Pancreatic cancer and two additional cases of any LS-associated cancer (Table 6) in the same person or in close relatives |
|  | ≥3 cases of pancreatic cancer and/or melanoma in close relatives |
|  | Pancreatic cancer and melanoma in the same person |
| Prostate cancer |  |
|  | ≥2 cases of prostate cancer dx at age ≤55 in close relatives |
|  | ≥3 FDRs with prostate cancer |
|  | Aggressive (Gleason score >7) prostate cancer and ≥2 cases of breast, ovarian, and/or pancreatic cancer in close relatives |
| Renal cancer |  |
|  | RCC with clear cell histology, if any of the following criteria are met:  –dx at age <50  –Bilateral or multifocal tumors  –≥1 close relative with clear cell RCC |
|  | RCC with papillary type 1 histology |
|  | RCC with papillary type 2 histology |
|  | RCC with collecting duct histology |
|  | RCC with tubulopapillary histology |
|  | RCC with BHD-related histology (chromophobe, oncocytoma, oncocytic hybrid) |
|  | Urothelial carcinoma (or transitional cell carcinoma) and 2 additional cases of any LS-associated cancer (Table 6) in the same person or in relatives |
| Thyroid cancer |  |
|  | Medullary thyroid cancer |
|  | Papillary thyroid cancer (cribriform-morular variant) |

Table S2. NCCN guidelines and criteria implemented: Criteria from breast, ovarian, pancreatic, and colorectal cancer CPGs from NCCN were represented with equivalency classes in the ontology.

| NCCN Hereditary Cancer CPGs implemented in CPG Ontology | |
| --- | --- |
| Breast, Ovarian, Pancreatic: CRIT-1 |  |
|  | Individuals with any blood relative with a known pathogenic/likely pathogenic variant in a cancer susceptibility gene |
|  | Breast cancer dx at age ≤45 |
|  | Breast cancer dx at age 46-50 y with an unknown or limited family history |
|  | Breast cancer dx at age 46-50 y with a second breast cancer dx at any age |
|  | Breast cancer dx at age 46-50 y with ≥1 close blood relative with breast |
|  | ovarian, pancreatic, or high-grade (Gleason score ≥7) or intraductal |
|  | prostate cancer at any age |
|  | Breast cancer dx at age ≤60 y with triple-negative breast cancer |
|  | Breast cancer dx at any age with Ashkenazi Jewish ancestry |
|  | Breast cancer dx at any age with ≥1 close blood relative with breast cancer at age ≤50 y or ovarian, pancreatic, or metastatic or intraductal prostate cancer at any age |
|  | ≥3 total diagnoses of breast cancer in patient and/or close blood relatives |
|  | Diagnosed at any age with male breast cancer |
|  | Epithelial ovarian cancer (including fallopian tube cancer or peritoneal cancer) at any age |
|  | Exocrine pancreatic cancer at any age |
|  | Metastatic or intraductal prostate cancer at any age |
|  | High-grade (Gleason score ≥7) prostate cancer with Ashkenazi Jewish ancestry |
|  | High-grade (Gleason score ≥7) prostate cancer with ≥1 close blood relative with breast cancer at age ≤50 y or ovarian, pancreatic, or metastatic or intraductal prostate cancer at any age |
|  | High-grade (Gleason score ≥7) prostate cancer with ≥2 close blood relatives with breast or prostate cancer at any age |
|  | An affected or unaffected individual with a first- or second-degree blood relative meeting any of the NCCN Genetic/Familial High-Risk Assessment: Breast, Ovarian, and Pancreatic personal risk criteria from CRIT-1 |
| Breast, Ovarian, Pancreatic: CRIT-4 |  |
|  | Individual from a family with a known TP53 pathogenic/likely pathogenic variant |
|  | Individual diagnosed at age <45 y with Non Ewing Sarcoma AND a first degree relative diagnosed at age <45 y with cancer AND an additional first- or second-degree relative in the same lineage with cancer diagnosed at age <45 y, or a sarcoma at any age |
|  | Individual with a tumor from LFS tumor spectrum (eg, soft tissue sarcoma, osteosarcoma, CNS tumor, breast cancer, adrenocortical carcinoma), before 46 y of age, AND at least one first- or second-degree relative with any of the aforementioned cancers (other than breast cancer if the proband has breast cancer) before the age of 56 y or with multiple primaries at any age |
|  | Individual with multiple tumors (except multiple breast tumors), two of which belong to LFS tumor spectrum with the initial cancer occurring before the age of 46 y |
|  | Individual with adrenocortical carcinoma, or choroid plexus carcinoma or rhabdomyosarcoma of embryonal anaplastic subtype, at any age of onset, regardless of family history |
|  | Breast cancer before 31 y of age |
| Breast, Ovarian, Pancreatic: GENE-1 |  |
|  | Meets NCCN Genetic/Familial High-Risk Assessment: Breast, Ovarian, and Pancreatic criteria from CRIT-1, CRIT-2, CRIT-4, or CRIT-5 and familial pathogenic/likely pathogenic variant known |
|  | Meets NCCN Genetic/Familial High-Risk Assessment: Breast, Ovarian, and Pancreatic criteria from CRIT-1, CRIT-2, CRIT-4, or CRIT-5 but no known familial pathogenic/likely pathogenic variant |
| Colorectal: HRS-3 |  |
|  | An individual with colorectal or endometrial cancer at any age with tumor showing evidence of mismatch repair (MMR) deficiency, either by microsatellite instability (MSI) or loss of MMR protein expression |
|  | Colorectal or endometrial cancer diagnosed <50 y |
|  | An individual with colorectal or endometrial cancer and another synchronous or metachronous LS-related cancer |
|  | An individual with colorectal or endometrial cancer and ≥1 first-degree or second-degree relative with LS-related cancer diagnosed <50 y |
|  | An individual with colorectal or endometrial cancer and ≥2 first-degree or second-degree relative with LS-related cancers regardless of age |
|  | ≥1 first-degree relative with colorectal or endometrial cancer diagnosed <50 y |
|  | ≥1 first-degree relative with colorectal or endometrial cancer and another synchronous or metachronous LS-related cancer |
|  | ≥2 first-degree or second-degree relatives with LS-related cancers, including ≥1 diagnosed <50 y |
|  | ≥3 first-degree or second-degree relatives with LS-related cancers, regardless of age |
| Colorectal: LS-1 |  |
|  | Meets HRS-3 criteria and LS pathogenic/likely pathogenic variant known in family |
|  | Meets HRS-3 criteria but no known pathogenic/likely pathogenic variant in proband or family |
| Colorectal: JPS-1 |  |
|  | ≥5 juvenile polyps of the colon |
|  | Any number of juvenile polyps in an individual with a family history of JPS |
| Colorectal: PJS-1 |  |
|  | Family history of PJS |
| Colorectal: POLYP-1 |  |
|  | Personal history of ≥20 cumulative adenomas, personal history of desmoid tumor, hepatoblastoma, cribriform morular variant of papillary thyroid cancer, or multifocal/bilateral congenital hypertrophy of the retinal pigment epithelium (CHRPE). Or if individual meets SPS1.01 or SPS3.03 criteria with at least some adenomas or personal history of between 11-20 cumulative adenomas |
| Colorectal: SPS-1 |  |
|  | At least 5 serrated polyps proximal to the sigmoid colon with 2 or more of these being >10 mm |
|  | Any number of serrated polyps proximal to the sigmoid colon in an individual who has a first-degree relative with serrated polyposis |
|  | ≥20 serrated polyps of any size, but distributed throughout the colon |

Table S3. Cancer concept identifiers: Cancer concept identifies within the ontology necessary to create an FHx JSON request object.

| Cancers in the CPG ontology | | | | | |
| --- | --- | --- | --- | --- | --- |
| name | conceptId | name | conceptId | name | conceptId |
|  |  |  |  |  |  |
| Cancer | IRF100173 | Skin cancer | IRF100146 | Osteosarcoma | IRF100086 |
| Multiple myeloma | IRF100147 | Sebaceous cancer | IRF100143 | Ewing's sarcoma | IRF100087 |
| Parathyroid cancer | IRF100148 | Basal cell skin cancer | IRF100144 | Appendix cancer | IRF100160 |
| Esophageal cancer | IRF100149 | Squamous cell skin cancer | IRF100145 | Cecum cancer | IRF100035 |
| Salivary gland cancer | IRF100152 | Melanoma | IRF100141 | Eye cancer | IRF100163 |
| Fallopian tube cancer | IRF100021 | Small bowel cancer | IRF100138 | Retinoblastoma | IRF100161 |
| Schwannoma | IRF100153 | Stomach cancer | IRF100132 | Ocular melanoma | IRF100162 |
| Vulva cancer | IRF100154 | Testicular cancer | IRF100156 | Leukemia | IRF100121 |
| Gallbladder cancer | IRF100155 | Thyroid cancer | IRF100136 | Acute lymphoblastic (ALL) | IRF100114 |
| Kidney cancer | IRF100067 | Medullary thyroid cancer | IRF100135 | Chronic lymphocytic (CLL) | IRF100115 |
| Wilms tumor | IRF100054 | Follicular thyroid cancer | IRF100133 | Acute myelogenous (AML) | IRF100116 |
| Lung cancer | IRF100126 | Papillary thyroid cancer | IRF100134 | Chronic myelogenous (CML) | IRF100117 |
| Squamous cell lung cancer | IRF100122 | Ureteral cancer | IRF100075 | Throat cancer | IRF100150 |
| Small cell lung cancer | IRF100123 | Brain cancer | IRF100108 | Anal cancer | IRF100034 |
| Lymphoma | IRF100053 | Choroid plexus cancer | IRF100100 | Mouth cancer | IRF100151 |
| Hodgkin's lymphoma | IRF100051 | Ependymoma | IRF100101 | Bladder cancer | IRF100071 |
| Non-Hodgkin's lymphoma | IRF100052 | Glioma | IRF100102 | Atypical teratoid rhabdoid tumor | IRF100164 |
| Ovarian cancer | IRF100019 | Medulloblastoma | IRF100103 | Colorectal cancer | IRF100044 |
| Pancreatic cancer | IRF100033 | Meningioma | IRF100104 | Colon cancer | IRF100036 |
| Primary peritoneal cancer | IRF100023 | Pinealoblastoma | IRF100105 | Rectum cancer | IRF100037 |
| Prostate cancer | IRF100029 | Hemangioblastoma | IRF100106 | Colon polyps | IRF100038 |
| Sarcoma | IRF100094 | Endometrial cancer | IRF100050 | Adrenal cancer | IRF100113 |
| Chondrosarcoma | IRF100079 | Cervical cancer | IRF100045 | Biliary tract cancer | IRF100140 |
| Leiomyosarcoma | IRF100078 | Liver cancer | IRF100159 | Chordoma | IRF100165 |
| Liposarcoma | IRF100080 | Hepatoblastoma | IRF100157 | Endolymphatic sac tumor | IRF100166 |
| Neurofibrosarcoma | IRF100076 | Hepatocellular carcinoma | IRF100158 | Breast cancer | IRF100015 |
| Rhabdomyosarcoma | IRF100077 | Bone cancer | IRF100088 |  |  |

Table S4. Gene concept identifiers: Gene concept identifies within the ontology necessary to create an FHx JSON request object.

| Genes in the CPG ontology | | | | | | | |
| --- | --- | --- | --- | --- | --- | --- | --- |
| name | conceptId | name | conceptId | name | conceptId | name | conceptId |
|  |  |  |  |  |  |  |  |
| TP53 | IRF100312 | SMC1A | IRF100299 | POLD1 | IRF100269 | GATA2 | IRF100226 |
| PMS2 | IRF100268 | SMC3 | IRF100300 | POLE | IRF100270 | HRAS | IRF100235 |
| ETV6 | IRF100215 | SMO | IRF100301 | TSC1 | IRF100307 | MSH6 | IRF100254 |
| EZH2 | IRF100216 | SRC | IRF100302 | TSC2 | IRF100308 | IDH1 | IRF100236 |
| FBXW7 | IRF100218 | SRSF2 | IRF100303 | MLH1 | IRF100250 | IDH2 | IRF100237 |
| FGFR1 | IRF100219 | STAG2 | IRF100304 | FH | IRF100222 | JAK3 | IRF100239 |
| FGFR2 | IRF100220 | TERC | IRF100309 | FLCN | IRF100223 | KRAS | IRF100244 |
| FGFR3 | IRF100221 | TERT | IRF100310 | MAX | IRF100246 | MPL | IRF100251 |
| FLT3 | IRF100224 | WARN | IRF100314 | RB1 | IRF100281 | NPM1 | IRF100260 |
| GATA1 | IRF100225 | PALB2 | IRF100263 | SDHA | IRF100286 | NRAS | IRF100261 |
| GNA11 | IRF100229 | CSF1R | IRF100203 | SDHAF2 | IRF100287 | PDGFRA | IRF100264 |
| GNAQ | IRF100228 | KDR | IRF100242 | SMARCA4 | IRF100296 | PTPN11 | IRF100276 |
| CDH1 | IRF100196 | MUTYH | IRF100255 | TMEM127 | IRF100311 | RUNX1 | IRF100285 |
| GNAS | IRF100227 | CHEK2 | IRF100202 | ALK | IRF100176 | STK11 | IRF100305 |
| GPC3 | IRF100230 | VHL | IRF100313 | MITF | IRF100249 | WT1 | IRF100315 |
| HMRNPK | IRF100232 | BMPR1A | IRF100186 | MSH2 | IRF100253 | ABL1 | IRF100174 |
| HNF1A | IRF100233 | CDKN2A | IRF100199 | RAD50 | IRF100278 | AKT1 | IRF100175 |
| HOXB13 | IRF100234 | PTEN | IRF100275 | AXIN2 | IRF100179 | ASXL1 | IRF100180 |
| JAK2 | IRF100238 | SMAD4 | IRF100295 | MRE11A | IRF100252 | BCOR | IRF100183 |
| KDM6A | IRF100241 | NBN | IRF100256 | NF2 | IRF100258 | BCORL1 | IRF100184 |
| KMT2A | IRF100243 | BARD1 | IRF100182 | PHOX2B | IRF100266 | BLM | IRF100185 |
| LUC7L2 | IRF100245 | BRIP1 | IRF100191 | SMARCB1 | IRF100297 | BRAF | IRF100187 |
| NOTCH1 | IRF100259 | CDK4 | IRF100200 | SUFU | IRF100306 | CALR | IRF100192 |
| APC | IRF100177 | RAD51C | IRF100279 | FANCC | IRF100217 | CASR | IRF100193 |
| NSD1 | IRF100262 | RAD51D | IRF100280 | XRCC2 | IRF100316 | EPCAM | IRF100212 |
| PHF6 | IRF100265 | SDHB | IRF100288 | CDC73 | IRF100195 | CBL | IRF100194 |
| PIK3CA | IRF100267 | SDHC | IRF100289 | BRCA1 | IRF100188 | CDKN1C | IRF100198 |
| PRPF40B | IRF100273 | SDHD | IRF100290 | DICER1 | IRF100206 | CSF3R | IRF100204 |
| RAD21 | IRF100277 | BRCA2 | IRF100189 | KIT | IRF100240 | CTNNB1 | IRF100205 |
| RECOL4 | IRF100283 | NF1 | IRF100257 | PRKR1A | IRF100272 | DIS3L2 | IRF100207 |
| SETPB1 | IRF100291 | BAP1 | IRF100181 | PTCH1 | IRF100274 | DNMT1 | IRF100208 |
| SF1 | IRF100292 | RET | IRF100284 | BRINP3 | IRF100190 | DNMT3A | IRF100209 |
| SF3A1 | IRF100293 | GREM1 | IRF100231 | CDKN1B | IRF100197 | EED | IRF100210 |
| SF3B1 | IRF100294 | MEN1 | IRF100247 | CEBPA | IRF100201 | ERBB2 | IRF100213 |
| ATM | IRF100178 | MET | IRF100248 | EGFR | IRF100211 | ERBB4 | IRF100214 |
| SMARCE1 | IRF100298 |  |  |  |  |  |  |
